# The updated AMSA scorecard of conflict-of-interest policies: a survey of U.S. medical schools

**DOI:** 10.1186/s12909-016-0725-y

**Published:** 2016-08-12

**Authors:** Daniel J. Carlat, Teddy Fagrelius, Reshma Ramachandran, Joseph S. Ross, Sallyann Bergh

**Affiliations:** 1 Tufts University School of Medicine, Boston, MA USA; 2 Johns Hopkins Hospital, Baltimore, MD USA; 3 Johns Hopkins School of Public Health, Baltimore, MD USA; 4 Yale University School of Medicine, New Haven, CT USA; 5 The Pew Charitable Trusts, Washington DC, USA; 6 P.O. Box 626, Newburyport, MA 01950, USA

**Keywords:** Conflict of interest, Policies, Medical schools, Pharmaceutical industry interactions

## Abstract

**Background:**

Best practices for conflict-of-interest (COI) policies in medical schools have evolved rapidly over the past decade, in part motivated by the American Medical Student Association (AMSA) scorecard that has publicly graded schools since 2007. This report describes the methodological update and impact of revisions to the scorecard in 2014.

**Methods:**

The original AMSA scorecard (used annually from 2008 to 2013) was revised by a work group to improve its methodology and to increase the stringency of its criteria for scoring COI policies. All U.S. medical schools (both allopathic and osteopathic; *n* = 160) were invited to submit their COI policies to AMSA for scoring with the new scorecard; web site searches were used to acquire policy information for schools that did not submit. The authors developed a codebook and analyzed 14 distinct categories of COI policies, pertaining to activities such as industry-funded gifts, meals, educational events, site access for sales reps, and conflict-of-interest disclosure requirements. The analysis yielded four possible grades for each school: A, B, C, or I (incomplete). The authors compared 2014 grades with 2013 grades, and compared the distribution of grades of schools by type (allopathic vs. osteopathic) and geographical region.

**Results:**

A total of 27 (16.9 %) medical schools scored A grades, indicating that their COI policies were strong, 81 (50.6 %) scored B, 25 (15.6 %) scored C and 26 (16.3 %) policies scored I. As compared to 2013, in 2014 fewer schools qualified for A grades (17.0 % vs. 26.0 %; *p* = 0.05). The grade distributions of allopathic and osteopathic schools were significantly different (*p* < 0.0001), with osteopathic schools more likely than allopathic schools to have incomplete policies. There were no significant grade differences by geographical region.

**Conclusions:**

The revised 2014 AMSA scorecard, with its more stringent criteria for evaluating COI policies, assigned fewer As and more Bs and Cs than in years past. This was the first study to identify schools with COI policies stronger than those recommended in 2008 by the Association of American Medical Colleges. Developing more stringent COI policies should be helpful in reducing the influence of pharmaceutical and device industry marketing on both trainees and faculty in American medical schools.

**Electronic supplementary material:**

The online version of this article (doi:10.1186/s12909-016-0725-y) contains supplementary material, which is available to authorized users.

## Background

While collaboration between academic physicians and universities and industry benefit medical research and has led to the development of many important treatments, they also have the potential to influence research and medical education [1]. Studies have shown that industry influence—whether in the form of gifts, commercially supported education, or simply visits with pharmaceutical representatives—can lead to more expensive and less evidence-based prescribing practices [2].

In response to these concerns, several national organizations recommended that medical schools create explicit guidelines for regulating the relationships between physicians and industry. Particularly influential were the recommendations published by the Association of American Medical Colleges (AAMC) which recommended the elimination, or strict regulation, of a variety of industry-funded activities, such as the provision of gifts and meals, continuing medical education (CME), speakers bureaus and the presence of sales representatives on campus [3].

In 2007, the American Medical Student Association (AMSA) began assessing medical schools’ conflict-of-interest (COI) policies by simply reporting whether or not schools had developed such policies. In 2008, AMSA collaborated with the Pew Prescription Project to create a more elaborate scorecard which analyzed 11 COI policy domains, such as industry-funded gifts and meals, educational programs, and scholarships. Schools were invited to submit policies directly to AMSA, which were analyzed on a four-point scale from 0 (no policy) to 3 (model policy). Schools which did not respond to requests for policies received an automatic F, while those reporting that their policies were under revision scored a provisional I for in process.

The scorecard assessed polices annually from 2008 to 2013 (the 2011–2012 scorecards were combined); by 2013, the percentage of medical schools receiving overall A grades had increased steadily to 25.9 %, from 4.7 % in 2008 [4]. Since its inception, the AMSA scorecard has generated considerable media attention and has influenced the development or strengthening of COI policies at many academic institutions [5–7]. This report describes the methodological update and impact of revisions to the scorecard in 2014.

## Methods

### Revision of AMSA scorecard

In 2011, The Pew Charitable Trusts and AMSA planned for a major revision of the scorecard, an effort that was supported by a grant from the Oregon Attorney General Consumer and Prescriber Education Program [8]. The purposes of the revision were to update the criteria for model policies and to enhance the instrument’s methodological rigor [9].

In order to accomplish this, two committees were created: an expert task force on medical conflicts of interest and a scorecard methodology work group. The expert task force, composed of leaders in academic medicine as well as other partners, including AMSA, met 5 times between May 2012 and January 2013. This committee reviewed the literature on COI, interviewed other experts about the feasibility of implementing policies in academic medical centers, and reviewed model policies currently in existence. By consensus the group adopted aspirational best practices, with the understanding that reaching these goals would take time for institutions, especially those with academic cultures resistant to such changes. The task force published its recommendations in December 2013 [10, 11]. In addition to the expert task force, a methodology work group was formed composed of representatives from Pew (DJC and SB), AMSA (TF and RR), and a methodology consultant (JSR). We (the methodology work group) met eight times from July 2012 to March 2014, examining and revising the domains used in the original scorecard, as well as the scoring system. Overall, three major changes were made to the AMSA Scorecard: the COI policy domains were revised and expanded, the criteria to score each domain was revised, and the formula used to aggregate the domain scores to calculate an overall grade was revised.

#### Revising and expanding COI domains

We increased the number of COI domains from 11 to 14 to better reflect the breadth of policies in medical schools (Table 1). Some domains were split into two categories, because medical schools have been developing policies that address them as distinct areas. For example, “gifts including meals” was split into “gifts” and “meals,” and “industry sales representatives” was separated into “pharmaceutical sales representatives” and “medical device representatives.” We added some new domains, such as “ghostwriting” and “extension,” the latter being a domain to assess whether faculty are required to abide by the medical school’s COI policies even when they practice or teach in other settings. While we did not eliminate any domains, we shifted two from the medical school scorecard to a new scorecard tailored specifically for teaching hospitals, namely, “pharmaceutical samples” and “pharmacy and therapeutics (P&T) committees”.

**Table 1:** Revision of AMSA scorecard model COI policies

Policy Area	2008 AAMC Recommendation	Original AMSA Scorecard Model Policy	2014 AMSA Scorecard Model Policy
Conflict of Interest Disclosure	Domain not addressed	Disclose on public website and/or to patients	Disclose to institution and externally to medical students and trainees
Industry-funded speakers’ bureaus^a^	Strongly discouraged	Prohibited	Prohibited
Industry-support of accredited CME	Permitted if funds coordinated and overseen by a central CME office	Permitted if funds coordinated and overseen by a central CME office	Permitted in rare circumstances such as blinded pool of funds not earmarked for specific courses
Attendance of unaccredited industry-sponsored educational events	Prohibited	Permitted, but travel funds must be awarded independent of industry influence	Prohibited
Access of pharmaceutical sales representatives to AMCs^a^	Permitted by appointment only, and restricted to non-patient areas.	Prohibited; non-marketing interactions permitted	Prohibited; non-marketing interactions permitted
Access of medical device representatives to AMCs^b^	Permitted by appointment, with prior disclosure and patient consent, and for technical assistance and in-service training only.	Domain not addressed	Permitted by appointment, with prior disclosure and patient consent, and for technical assistance and in-service training only.
COI curriculum^a^	Required for all phases of medical education.	Required for all phases of medical education.	Required, with a comprehensive curriculum mirroring AMSA best practices.
Extension of AMC COI policies to community teaching affiliates and to all faculty^b^	Required off-site.	Domain not addressed	Required off-site and for all faculty, including voluntary.
Gifts	Prohibited	Prohibited except textbooks	Prohibited
Meals	Prohibited, except when in connection with CME programming.	Prohibited, except when in connection with CME programming.	All meals prohibited, including in CME courses
Consulting	Permitted if compensation reflects the fair market value of the services provided.	Permitted with prior approval, plus either formal contract or payment commensurate to deliverable	Permitted if consulting is limited to scientific and not marketing topics.
Ghostwriting and Honorary Authorship^b^	Prohibited	Domain not addressed	Prohibited
Industry-Supported Travel Scholarships for Trainees (excluding support for research training)	Permitted, but recipient selection must be independent of industry sponsor	Permitted, but recipient selection must be independent of industry sponsor	Prohibited
Enforcement^b^	Domain not addressed	Required, but not part of formal scoring	Required, and integrated into final score

^a^Model policies identical in original and new versions

^b^Policies not present in original version of scorecard

#### Revising the criteria for scoring domains

We revised the criteria used for scoring policies such that most domains were scored more stringently (see Table 1 for a comparison of model policies in the two scorecards). These changes were based on the recommendations of the expert task force as well as other sources, such as the revised American Medical Association ethics policy regarding industry funding of CME [12]. In addition, we altered some ratings so that they were better aligned with reporting requirements of the Physician Payments Sunshine Act [13]. For instance, the Sunshine Act requires that the value of all meals paid for by industry at CME courses be reported. Accordingly, the scorecard required prohibition of industry supported CME meals in order to achieve the highest score in the meals category.

We assigned model policies a score of ‘3’. Non-model policies were scored as either a ‘2’ (defined as good progress toward model policy) or a ‘1’ (defined as policy that does not address this domain or is unlikely to have a substantial effect on behavior). We developed a scoring codebook (available on AMSA’s Scorecard website) that included each COI policy domain and examples of policies that would score a 1, 2, or 3 for each domain [14]. In contrast to the original AMSA scoring system, which was a 4 point system (0–3), the new system is a 3 point system (1–3), with non-existent policies scored as “1”, identical to the score for ineffectual policies.

#### Revising the scorecard grading formula

We revised the original formula used to calculate overall grades in two ways. First, we simplified policy scoring by weighting all 14 domains equally, rather than weighting some domains more heavily than others, as the original scorecard had done. Second, we eliminated the D and F grades since it was not clear that distinguishing among 5 different calibers of policies was necessary or meaningful.

We derived a total score for each medical school by adding the individual scores for each of the 14 domains. Since each domain can score from 1 (poor or no policy) to 3 (model policy), the possible scoring range for each school was 14–42. Based on the raw score, we calculated a standardized score with the following formula: (Raw score * 2) + 16 = Standardized score. We used this formula in order to convert the maximum raw score of 42 into a more intuitive maximum of 100. To transform the standardized score into a percentage, we divided it by the maximum score and multiplied by 100. Finally, we created rules for assigning percentages into letter grades (Table 2).

**Table 2:** Methodology for assigning letter grades to medical schools’ COI policies

Letter grade	Description	Percentage score	Corresponding raw score
A	For a school to achieve this grade, at least half (7 of 14) of its COI policies were rated as excellent.	≥ 85 %	≥ 35
B	Up to 6 COI policies were excellent.	≥ 72 %.	28–34
C	Up to half of policies were poor or absent.	≥ 56 %	20–27
I	More than half of policies were poor or absent.	≤ 54 %	≤ 19

### Regrading medical school COI policies

In order to collect information on medical school COI policies for the revised AMSA Scorecard, we invited all U.S. schools of allopathic and osteopathic medicine to submit policies to AMSA for grading. We sent emails to appropriate contacts at these schools (deans, COI officers, etc. as established by previous year’s contact at each school) explaining the Scorecard project and requesting the submission of their policies (Additional file 1).

For those schools that did not respond to our request for submissions, we searched online for their COI policies. In order to maximize our ability to retrieve all policies, we developed a systematic search process. This included using a list of search terms (such as “conflict of interest,” “industry interactions,” and “vendor policies”), searching the prior AMSA records for any publically posted policies, and checking the Institute on Medicine as a Profession (IMAP) website, which publishes a database of publically available policy documents [15]. When we could not find information on particular domains, we emailed and called compliance officers to ask about specific policies. In cases where we were unable to retrieve policies for specific scoring areas, we noted “no policy found” in our documentation.

Prior to re-grading the medical school COI policies, all analysts were provided with formal training in the revised scoring system and use of the scoring codebook. We assessed the reliability of our scoring system by having all analysts score the policies of five medical schools. Our inter-rater reliability, measured using percent agreement, varied from 80.0 to 92.0 %, with Kappa scores ranging from 0.65 to 0.85, across the four analysts.

In addition, because we used two different methodologies to retrieve policy information (assessing submitted policies submitted vs. finding policies via web-searches), there was a possibility of bias in favor of those schools which submitted policies. Web searches may miss COI policies that exist, but which institutions have not made publically available. This, in turn, could lead to falsely low grades for such schools. In order to assess the robustness of our web-search methodology, we randomly selected 15 % of the schools which had submitted complete policies, and rescored them using policies identified from web-searches. In 9 out of 10 of these schools, the final grade did not change, validating our use of both submitted and web-search identified COI policies for the 2014 AMSA Scorecard.

### Statistical analysis

We used descriptive statistics to characterize the COI policies identified for medical schools, overall and stratified by two key characteristics: type of school (allopathic vs. osteopathic) and location (categorized by U.S. Census Regions: northeast, south, midwest, west). In addition, we used chi-square and Fischer Exact tests to examine differences in overall AMSA Scorecard grades in 2013 and 2014, as well as for differences in 2014 grades by the medical school characteristics described above. All analyses were conducted using JMP 10.0 (SAS Institute; Cary, NC). All statistical tests were two-tailed and used a type I error rate of 0.017 to account for multiple comparisons across 3 medical school characteristics.

## Results

### Overall grades

Our call for COI policies yielded submissions from 77 of 161 medical schools, a 47.8 % response rate. We scored the remainder of schools based on policies identified via web searches. Overall, school grades were as follows: 27 received A (16.9 %), 81 B (50.6 %), 25 C (15.6 %), and 26 Incompletes, or I (16.3 %) and represented a significantly different distribution in 2014 when compared with 2013 (*p* < 0.001; Table 3). In particular, consistent with the more stringent scoring criteria, fewer schools received A’s in 2014 when compared with 2013 (17.0 % vs. 26.0 %; *p* = 0.05).

**Table 3:** Distribution of medical school COI scorecard grades, 2013 vs. 2014

Overall COI Scorecard Grade	2013 Scorecard Grading (*n* = 158)	2014 Scorecard Grading (*n* = 160)
A	41 (25.9 %)	27 (16.9 %)
B	74 (46.8 %)	82 (50.6 %)
C	13 (8.2 %)	25 (15.6 %)
D	13 (8.2 %)	N/A
F	11 (7.0 %)	N/A
Incomplete or In process	6 (3.8 %)	26 (16.3 %)

*p* < 0.001for overall distribution of grades

### Individual domains

Because the criteria for ratings of individual COI domains varied between 2013 and 2014, direct comparisons for most domains are not meaningful, with two exceptions: speakers’ bureaus and pharmaceutical representatives (Table 4). In all years of the AMSA scorecard, the model policy was to forbid faculty from participating in promotional speakers bureaus, and to ban promotional detailing by pharmaceutical representatives.

**Table 4:** Frequency of model COI policies by individual domains, 2013 vs. 2014

COI Domain	Model COI Policies, No. (%)^a^		*p*
	2013 Scorecard Grading	2014 Scorecard Grading	
	(*n* = 158)	(*n* = 160)	
Disclosure	39 (24.7 %)	51 (31.7 %)	0.15
Speakers’ bureaus^b^	43 (27.2 %)	79 (49.4 %)	< 0.0001
Continuing medical education	101 (63.9 %)	5 (3.1 %)	< 0.0001
Attendance of promotional events	N/A	25 (15.6 %)	n/a
Pharmaceutical sales representatives^b^	4 (2.5 %)	9 (5.6 %)	0.26
Medical device representatives	N/A	91 (56.9 %)	n/a
COI curriculum	81 (51.3 %)	34 (21.3 %)	< 0.0001
Extension of COI policies	N/A	50 (31.3 %)	n/a
Gifts^c^	93 (58.9 %)	79 (49.4 %)	0.09
Meals^c^	93 (58.9 %)	24 (15 %)	< 0.0001
Consulting	71 (44.9 %)	26 (16.3 %)	< 0.0001
Ghostwriting	N/A	105 (65.6 %)	n/a
Scholarships	121 (76.6 %)	3 (1.9 %)	< 0.0001
Enforcement	N/A	126 (78.8 %)	n/a

*Notes*: ^a^Model policy defined as scoring a ‘3’ within the AMSA Scorecard codebook

^b^COI domains with model criteria that were identical in 2013 and 2014; all other criteria for model policies were changed in 2014

^c^Gifts and meals were scored as one domain, “gifts”, in the 2013 version

In 2014, 79 schools (49.4 %) effectively banned their faculty from serving on industry promotional speaker’s bureaus, up from 43 (27.2 %) in 2013. In addition, 9 schools (5.6 %) banned pharmaceutical detailing in 2014, up from 4 schools (2.5 %) in 2013. For several domains, dramatically fewer schools were rated as having model policies. Examples include meals (15 % in 2014, down from 58.9 % in 2013), CME (3.1 %, down from 63.9 %), scholarships (1.9 %, down from 76.6 %), consulting (16.3 %, down from 44.9 %), curriculum (21.3 %, down from 51.3 %), and gifts (49.1 % in 2014 versus 58.9 % in 2013) (Table 4).

The COI domains with the highest number of medical schools having model policies were speakers’ bureaus (79; 49.1 %), medical device representatives (91; 56.5 %), gifts (79; 49.4 %), ghostwriting (105; 65.6 %), and enforcement (126; 78.8 %).

### Comparisons of types of schools

We compared the grade distributions of schools by both medical school type and by geographic region (Table 5). We found that a significantly higher proportion of allopathic schools received A grades, while osteopathic schools were more likely to have incomplete policies (*p* < 0.001). All four geographic regions (northeast, south, west, midwest) had similar grade distributions. Schools from Puerto Rico were classified as “other.”

**Table 5:** 2014 AMSA scorecard grade, stratified by medical school characteristics, 2014

Medical School Characteristics	Overall AMSA Scorecard Grade				*P* value
	A	B	C	I	
*School Type (n = 160)*					< 0.0001
Allopathic (136 schools)	24 (17.6 %)	79 (58.1 %)	23 (16.9 %)	10 (7.4 %)	
Osteopathic (24 schools)*	3 (12.5 %)	3 (12.5 %)	2 (8.3 %)	16 (66.7 %)	
*School Location (n = 160)*†					0.20 (does not include the Puerto Rico schools)
Northeast *(39 schools)*	7 (17.9 %)	22 (56.4 %)	4 (10.4 %)	6 (15.4 %)	
South *(58 schools)*	11 (19 %)	28 (48.3 %)	10 (17.2 %)	9 (15.5 %)	
Midwest *(36 schools)*	4 (5.6 %)	18 (50 %)	11 (30.6 %)	3 (8.3 %)	
West *(23 schools)*	5 (21.7 %)	14 (60.9 %)	0 (0 %)	4 (17.4 %)	
Other *(Puerto Rico, 4 schools)*	0 (0 %)	0 (0 %)	0 (0 %)	4 (100 %)	

*Notes*: *Osteopathic medical schools that have not yet graduated a medical school class were not included in the 2014 Scorecard

^†^United States Census Bureau, “Census Regions and Divisions of the United States,” accessed May 26, 2016, https://www.census.gov/geo/reference/gtc/gtc_census_divreg.html

## Discussion

In 2014, AMSA revised its COI scorecard by streamlining its methodology, and making the criteria for model policies more stringent. Not surprisingly, fewer schools achieved an overall A grade on the COI scorecard in 2014 (16.9 %) than in 2013 (25.9 %). It is important to highlight that the decrease in schools with A grades does not imply that schools’ policies have become weaker since 2013, but rather reflects the strengthening of AMSA’s criteria for evaluating model policies. We set the policy bar higher to reflect a building consensus that the ideal medical education environment should be based entirely on current medical evidence, with no influence—whether direct or indirect—from the marketing departments of industry.

For five policy domains, we found that our revised criteria led to particularly dramatic reductions in the percentage of schools qualifying with model policies. Those domains are outlined below along with a brief explanation of why schools’ ratings decreased so profoundly.

1. Continuing medical education (63.9 % rated model policy in 2013, versus 3.1 % in 2014). The 2013 criteria reflected the 2008 AAMC recommendations that industry funding of CME is acceptable as long as all such funding is overseen by a central CME office in the medical school. Indeed, in 2013, the majority of schools had adopted this recommendation. However, the Pew Expert Task force recognized that central oversight, per se, is not always effective at preventing excessive reliance on industry CME funding, and that more stringent measures were needed. Accordingly, our Work Group revised the model criteria to align with these recommendations. The substantial decrease in schools with the model policy reflects the fact that schools are not ready to take measures to significantly reduce reliance on industry funding for CME.
2. COI Curriculum. (51.3 % rated model policy in 2013, versus 21.3 % in 2014). The original criteria defined a model policy as one that required some type of curriculum teaching the principles of COI for all phases of medical education (including at both medical schools and during residency training). The updated criteria went one step further, requiring that schools adopt a comprehensive curriculum reflecting AMSA best practices [16].
3. Meals (58.9 % rated model policy in 2013, versus 15 % in 2014). The AAMC and the original AMSA criteria allowed industry-funded meals only in the context of accredited CME events. However, the Pew Expert task force could find no evidence that meals paid for by industry at CME courses were any less likely to engender gratitude in the recipient than meals in other settings. In all cases, the recipients know who is paying for their food, and are therefore equally motivated to return the favor in some way. Recognizing this source of potential influence, the Physician Payments Sunshine Act requires public disclosure of all industry meals, even those at CME courses. Best COI practices would prohibit acceptance of any industry-funded meal; however, we found that only 15 % of schools had such a policy.
4. Consulting (44.9 % rated model policy in 2013, versus 16.3 % in 2014). The 2013 AMSA model policy criteria allowed consulting with industry as long as the consultant received permission in advance from their institution. The Pew expert task force recommended that medical schools limit faculty consulting to scientific topics only, and prohibit marketing consultation oriented toward product promotion. Using these criteria, we found that such robust policies were uncommon.
5. Scholarships (79.6 % rated model policy in 2013, versus 1.9 % in 2014). The 2013 criteria allowed industry to fund travel scholarships to trainees as long as the recipients were selected independently from the sponsor. The Pew expert task force reasoned that such scholarships were essentially gifts to trainees and were therefore likely to influence attitudes in favor of the funding company. Therefore, AMSA best practices suggest a prohibition of these scholarships, unless they are given to fund legitimate research activities. We found that such restrictive policies were very rare.

In contrast with the above examples, one domain—speakers’ bureaus—saw a significant rise in the proportion of schools with model policies, from 27.2 % in 2013 to 49.4 % in 2014. Since the scoring criteria were identical in the two versions of the scorecard, we can be confident that this change represents a genuine improvement in schools’ policies. Over the last several years, websites have publically disclosed the names of physicians who have received payments for promotional speaking, and this rise in transparency may have influenced schools to limit such activities to prevent negative publicity.

Our finding that osteopathic schools are significantly less likely to have complete COI policies may reflect less of a perceived need for such policies. Osteopathic schools are less likely than allopathic schools to own their own hospitals and to engage in clinical research [17], and we assume that this creates a natural barrier, leading to less contact with industry representatives. Many stakeholders (including accrediting and funding agencies, medical school applicants, and residency programs) are attentive to allopathic school-osteopathic school similarities and differences at many levels [18], and our findings contribute to that literature.

The Institute on Medicine as a Profession (IMAP), like us, has systematically evaluated medical school COI policies. Comparing 2008 with 2011, they found that the proportion of policies rated “moderate” increased from 18 to 72 %, but that there were very few schools with “strong” policies—1 % in 2008, and 4 % in 2011. IMAP characterized policy changes as a “race to the middle” rather than a race to the top [19]. It is not possible to compare our findings to those of IMAP because of differences in methodology, such as variations in model policy criteria.

We found that while many schools have imposed restrictions on certain financial relationships between physicians and companies, a culture of dependence on industry largesse—albeit a culture that is changing for the better—persists. For example, many schools ostensibly have no-gift policies, and yet most of them still allow companies to purchase expensive textbooks for students and faculty. Although pharmaceutical representatives are now typically banned from purchasing meals for academic staff, most schools still allow company gifting of meals when the payment is indirect, such as during industry supported CME courses. Similarly, while many schools forbid industry from paying faculty to attend meetings, the practice of allowing industry to fund “scholarships” to pay for students to attend conferences persisted in nearly all the schools we assessed.

Such payments have no clear benefit in terms of advancing medical research or patient treatment. Instead, they are likely to produce a sense of gratitude and reciprocity toward benefactor companies in both trainees and faculty—which may translate into clinical decisions favoring newer and more expensive drugs over equivalent and cheaper alternatives. Indeed, evidence has accumulated that medical schools with stronger COI policies graduate students who prescribe more rationally [20].

## Conclusion

The revised 2014 AMSA scorecard is unique in that it identifies which medical schools have implemented policies that are more stringent than those recommended by the Association of American Medical Colleges in 2008. We hope that these results will encourage schools to develop stronger COI policies in order to protect the integrity of medical education, as well as to protect the reputations of their institutions. This is important in light of the 2014 launch of the Open Payments website (mandated by the Sunshine Act), which publishes the names of most physician recipients of industry gifts, along with the dollar value of the gift, the brand name of the product related to the gift, and other data. We speculate that this comprehensive public disclosure may drive medical schools to reevaluate whether the relatively small financial benefits of items such as free textbooks and CME meals is worth the scrutiny of patients who may question whether their doctors are influenced by such gifts. The AMSA scorecard is publically available [21], and we anticipate that medical school leadership will use it to access examples of model policies as they develop stronger COI policies in the future.

## Additional file

Additional file 1: AMSA Scorecard Announcement. This is the text of an email announcing the overhaul of the AMSA Scorecard and requesting medical school conflict-of-interest policies for submission to the 2013–2014 Scorecard. It was sent from the AMSA Scorecard Director to individuals working in the field of compliance at medical institutions across the country. (DOCX 12 kb)

## Abbreviations

AAMC: Association of American Medical Colleges
AMSA: American Medical Student Association
CME: Continuing Medical Education
COI: Conflict Of Interest
IMAP: Institute on Medicine as a Profession.
