# Supplementary material for: The updated AMSA scorecard of conflict-of-interest policies: a survey of U.S. medical schools
Source: BMC Med Educ. 2016 Aug 12;16:202. doi: 10.1186/s12909-016-0725-y (PMC4983088; doi:10.1186/s12909-016-0725-y)
Supplement: Additional file 1: — AMSA Scorecard Announcement. This is the text of an email announcing the overhaul of the AMSA Scorecard and requesting medical school conflict-of-interest policies for submission to the 2013–2014 Scorecard. It was sent from the AMSA Scorecard Director to individuals working in the field of compliance at medical institutions across the country. (DOCX 12 kb) [file 12909_2016_725_MOESM1_ESM.docx]

**APPENDIX**

**APPENDIX 1: AMSA Scorecard Announcement**

*(Email to medical schools and teaching hospital compliance contacts, August 26, 2013)*

Dear Administrator,

In response to the large body of evidence showing that conflicts of interest between the medical profession and industry can negatively influence patient care, the American Medical Student Association (AMSA) launched the PharmFree Scorecard in 2007. The Scorecard is designed to be a tool for change and offers a framework for evaluating and strengthening conflict of interest policies at medical schools across the country. The Scorecard is possible thanks to your participation, and I am writing today to formally request your policies on relationships with the pharmaceutical and device industries for the 2013-2014 iteration of the Scorecard.

In the past year the Scorecard has undergone a major overhaul. Here are some highlights:

-grading domains and methodologies were revised

-we are expanding to include teaching hospitals

-policy submissions are now accepted online through a secure website

-our submission form was shortened based on your feedback

Please click here to submit your policies and find information on the newly revised Scorecard. (link: http://www.amsa.org/scorecard )

**We are asking every school to submit their policy documents via this link even if you have done so in the past. This is to ensure our files are up-to-date.**

Do not hesitate to contact me with any questions or concerns.

Thank you.

Brigitte Frette, Scorecard Director

Please note that while I have made every effort to ensure our contact database is current, your assistance in linking me to the correct person at your school for Scorecard matters is greatly appreciated.
